# Editorial and Miscellaneous

**Published:** 1856-01

**Authors:** 


### EDITORIAL AND MISCELLANEOUS.

### TO OUR FRIENDS.

Far from accomplishing what we have desired, in the way of furnishing a journal worthy of patronage, and efficient in the advancement of science, we still feel that there is every thing to encourage us in our efforts, and to stimulate to a higher aim and greater exertion, in the numerous and increasing assurances of interest, which we are receiving.

While it has been from the commencement, a settled purpose, with the projectors of this Journal, that its publication should be permanent, without regard to the amount of patronage—if there had been any doubt of their ability to carry out their design, it has been removed, beyond contingency, by the already sustaining list of subscribers, which we have upon our books.

We think it a matter of some importance to refer to this subject, from the fact that some are objecting to subscribing, and especially to paying the subscription in advance, from the apprehension that this Journal may go the way of its predecessors in the City of Atlanta. We can assure those who feel inclined to aid us in establishing it upon the basis of patronage, and thereby put it in our power to make it much more valuable, that it shall not be discontinued, and they may send on their names and subscriptions without any fear of such a result.

We now consider ourselves under renewed and more sacred obligations to redeem the promise with which we set out, and while we have been often dissatisfied with our own want of fitness for the conduct of such a publication as we desire to make it, and frequently compelled to contemn our own labors, as our friends have not "despised the day of small things," we take courage, hoping to present them something more worthy of their kindness and support in the future.

That we have not at all times succeeded in adapting our views and action to every one who has lent us a helping hand, is doubtless true, and the utter impossibility of succeeding in this, is of course apparent, and shall not be attempted upon our part.

While courtesy and kindness is extended to all, we profess to have some settled views and principles to guide us, in this, as in the other affairs of life, and though it is, of course, our object to retain our friends and make enemies of none, we expect to manifest that independence of thought and action, by which alone we can expect to deserve, secure or retain respect and confidence.

We have no reason to doubt that this course will be sustained, and our views are thus expressed from reflections arising out of a survey of the many difficulties which beset the path of an Editor—and which no one can well appreciate who has not been placed in such a position—rather than from any cause of complaint connected with the reception of our services. And now, at the commencement of a new year, we hope that our acquaintance will justify the solicitation for a more intimate and mutually binding relation, permitting us to regard you as identified personally with our success, promising more assiduous labor for the faithful performance of the trust committed to us, and requesting upon your part, a continuance of the generous indulgence heretofore extended—not failing to give us your aid in the way of contributions and *subscribers*.

---

**"DISCRETION IS THE BETTER PART OF VALOR."**

From the *history* of some individuals, we should suppose that they drew some consolation from the reflection that

"He who fights and runs away
May live to fight another day."

And to one* who has made a misstatement of facts, (whether intentional or not, we leave for his decision,) and failed to meet his own issue in a manly and direct manner, but has skulked away under cover of an abortive attempt at satire, "*we have no reply*," and shall not pursue him in his *usual* inglo-

*Assistant Editor of the Nashville Journal.

rious retreat, taking his own suggestion as true, that the *game* has been greatly overrated, and is really not worth the ammunition.

---

### BIBLIOGRAPHICAL.

We have received the "Pronouncing Medical Lexicon, containing the correct pronunciation and definition of most of the terms used by speakers and writers on medicine and the collateral sciences, with Addenda, by C. H. Cleaveland, M. D., member of the American Medical Association, &c.," published by Longly Brothers, 168½ Vine St., above fourth, Cincinnati. Without entering into the discussion which seems to be going on in reference to its originality, we have no hesitation in saying, that it is, in itself considered, an exceedingly valuable little work.

We have also received the Introductory to the Course of Lectures in the St. Louis Medical College, by Chas. A. Pope, A. M., M. D.; that of Professor Jno. C. Dalton, Jr., M. D., in the College of Physicians and Surgeons, New York; and one by Jno. R. Allen, M. D., at the opening of the course in the Medical Department of the Iowa State University, for which we return our thanks, and give some extracts from each. Professor Pope says:

"Man, physically speaking, is the perfection of backbones, and includes all beneath him. His fins and wings and paws, are arms and hands destined to obey the behests of the most developed of nervous centres. His capital vertebræ constitute a skull fit to contain the maximization of the brain, that of a Cuvier or a Webster. His facial angle, unlike that of the crocodile, the albatross, the dog, and the chimpanzee, approximates the retangular measure, and that adopted by the ancient Greek artists (100°) as their beau ideal of the beautiful and the intellectual.

"What endless varieties may be produced by a few elements! The twenty-six letters of the alphabet constitute all the libraries of the world, with their poesies, philosophies, histories and logics. The material universe itself, with all its variety of sea and land, of cloud and sunshine, of day and night, of dark forest and cultivated plain, of metals, salts and earths, of plants and animals, of bleak desert and smiling vales of Tempe and of Sharon, is the result of the varied combinations of some sixty-two or sixty-three simple substances. A few vertebræ, with their processes varied, constitute the entire vertebrate series, from the tiny minnow through all the range of whales and sea monsters, of lowly creeping things and lions, and hippopotami, up to the lord of all, man himself. Thus, by a few varying strokes of the chisel, the block of marble becomes a satyr or an Apollo Belvidere, a sphynx or a Farnese Hercules. Thus, by a few touches of his pencil, the painter converts the melancholy cast of features into a smile—the face of a man into that of a donkey. So, by various departures from the archetype, the animal is fitted to cleave the liquid element, to crawl or swim, to walk or leap upon the surface of the earth, or to soar aloft in the atmosphere. Thus, by a few variations in the vertebræ and their processes, the living being is a fish or a fowl, a snake or a turtle, a mouse or a mastodon, a monkey or a man."

"Yet, notwithstanding man as a vertebrate ranks with the fishes and the reptiles, the beasts of the forest and fields, and the birds of the air, he differs from them all, widely, in species and genus, and even order. From the Chimpanzee, which approaches him more closely than any other animal, he is separated by an impassable gulf. He is not only different in genus, but different in order, from the highest and most intelligent of mammals. The Gorilla, whose skull and brain somewhat resemble his, is vastly inferior in the evolume of brain, and is quadrumanous. Man alone is biped and bimanous. He is the perfection of the vertebrate development. He alone walks erect and looks up to heaven as a suppliant, and abroad over the earth as a lord. He is the realization of the great idea shadowed forth in the creations of former periods—the culminating point in the material universe—the end and aim of the archetypal plan of the Creator.

"Man is the sole species of his genus, the sole representative of his order. If in his physical organization he ranks with the beasts that perish, in his intellectual and moral faculties he is only a little lower than the angels. Possessing a form of unrivalled majesty and beauty, "a front like Jove, to threaten and command—a station like the herald Mercury, new lighted on a heaven kissing-hill," he moves without a peer, the paragon of animals."

Prof. Dalton says, in reference to physiological investigations: "That we cannot foretell, from our knowledge of the chemical reactions of a substance outside the body, what will be its reactions in the body; since the conditions under which it is placed are new.

"Ferrocyanide of potassium and perlactate of iron, by mutual decomposition, produce Prussian blue. But if the lactate of iron, be injected into the right jugular vein of an animal, and ferro-cyanide of potassium into the left, so that they may meet in the blood, no Prussian blue is produced. The serum of the blood holds both salts in solution, and yet they do not act on each other; because there exists also in the serum an organic substance, which by its presence prevents their usual reaction. If this organic substance be destroyed by a few drops of sulphuric acid, then the two salts are at liberty to act on each other, and the Prussian blue is immediately produced.

"If a solution of cyanide of mercury be injected into the femoral artery, it returns unchanged by the femoral vein, and the animal suffers no inconvenience. But if injected into the vein, and carried through the heart to the lungs, it destroys life in less than a minute; because in the tissue of the lungs it meets with a substance by which it is decomposed with the production of hydrocyanic acid, that poisons at once the nervous system, and stops the action of the heart.

"Such facts as these give us an idea of the peculiar delicacy and complexity of the phenomena which we meet with in studying Physiology. Singular as it may seem, there is a tendency in some minds to ignore this complexity in the phenomena of life,—to push it out of the way, even, and cover it up, as if it were a stumbling-block in the path of science, instead of being, as it is, an essential fact, to be recognised and studied like any other. I could name more than one physiological writer, whose whole endeavor seems to be to reduce the science, as it were, by force of arms, to a series of simple propositions, which do not express its real character. They attempt to square physiology on the pattern of other sciences, instead of taking it as it really presents itself, and in studying it as chemistry and physics, they forget to study it as physiology. A single example will make my meaning in this respect more easily understood. It is well known that a certain amount of sugar is constantly introduced into, or produced in the body; and that, as it enters the blood, it is destroyed and disappears as sugar, passing through a series of transformations, the details of which are not altogether understood. In Diabetes, this sugar, for some cause or other, is not destroyed as it is in health, and accumulates in the blood—making its appearance, consequently, in some of the secretions. Some years ago, the chemist Mialhe observed that when sugar was boiled with a solution of potass, it was destroyed under the influence of the alkali, losing the properties of sugar and becoming converted into a brown substance, known as melassic acid. Observing also that the serum of the blood was alkaline, he concluded that it was by this alkali that the sugar was naturally destroyed in the circulation; and that when the alkalescence of the serum, from any cause, was insufficient, all the sugar could not be destroyed by it, and therefore accumulated, producing the condition of Diabetes. He observed, in this instance, what took place in the test-tube, and from that inferred what took place in the blood—forgetting, by some inconceivable fatuity, the essential difference between a solution of caustic potass, at the temperature of 212°, and the slightly alkaline blood, composed of twenty different ingredients, at the temperature of 100°, and circulating in the vessels of the living body. His conclusion was worthless, as the expression of a physiological fact, for the simple reason that, in the body, the sugar is *not* boiled with caustic potass, but is subjected to other influences. The destruction of sugar by boiling potass, on which he based his theory, is a purely chemical fact, of a certain degree of importance, and extremely interesting to know; but it is not a physiological fact, and he was not, as he supposed, studying Physiology.

"Let us not, then, commit the mistake too commonly made, of taking it for granted that things will be in the body as they are in the test-tube and crucible. We cannot tell whether they will be so or not, until we look and see. If we persist in regarding the organised frame as a furnace or a filtering-jar, and its actions as identical with combustions and filtrations, we may amuse ourselves with introducing into Physiology an imaginary simplicity, but we shall make no progress in positive knowledge. If we wish to study the structure and growth of sea-weed, we do not look for it in fresh water. If we wish to study the functions and phenomena of life, we must search for them in the living body, and in the living body alone—take them as they are, and not compare them with other things which are dissimilar."

Professor Allen, in speaking of the ten thousand agencies fruitful in diversifying the human body, in its relation to the use of remedies, remarks: "Think you, yon diminutive, frail and suffering infant, born in the confines of a city, of a fashionable mother—deformed by stays, enfeebled by indolence, softened by luxury, torpid from inactivity, and etiolated from shunning the genial sun light, identical in physical development and vital power, with the offspring of the vigorous country housewife, ruddy with health, and ripe in development?

"Think you, yon poor child with waxen skin, pearly eye, fragile form, and feeble motion, equal in bodily stamina, to the village boy, "with shining morning face,"—fat, rotund and active?

"Think you, yon pale, slender and stooping young man toiling in the cavernous shades of the counting-house, or in the dust and smoke of a confined work-shop, to be compared in bodily strength and vital force with the sturdy yeoman, developed by salubrious toil, or the hardy mountaineer, ripened into manly stature and elastic action by the stirring chase and mountain breezes?

"Think you that the man of wealth, and effeminate ease, resists with equal power, morbific agents, with the sturdy peasant who pays his rents?

"Think you that the man of busines, absorbed in scenes of speculation, or harrassed by the perplexities of diversified transactions, equally exempt from disease or active in its combat, with the quiet agriculturalist who waits in sober industry for seed time and harvest to reward his toils?

"Think you the professional man, confined to his books and briefs, incessant study; or, as with him of our own self-sacrificing avocation, exposed to breath of charnal house, to heat and cold, harrassed by annoying thoughts and active toil, who is seen to fade, so early, into the sear and golden leaf, comparable in brawny development, or bodily powers, with the mechanic who by regular labor and alternate rest, sustains, to three-score years and ten, his healthy functions?

"To these peculiarities, evolved by the natural epochs of our lives, or induced by habits in conflict with natural laws, we must add those strongly marked, yet infinitely intermixed constitutional stamps, known as temperaments: distinguished by a predominance, more or less distinct, of some one of the systems or tissues of the organism.

"These, when very well defined, stamp impressively their own characteristics, or if mingled, produce infinite diversity of organic susceptibilities, or tinge with infinite variety the features of disease, and affect in unknown ways, the action of remedies.

"But still we must superadd to this long list those hereditary maladies arising in those unfortunate persons, in whom nature, by some unknown departure from her generally beneficent care, or as a lasting infliction upon the licentious and bestial, combines elements, which generate, unhappily, those physical ills transmissable from parent to child.

"Rheumatism thus prolongs its pains and fever—tortures its victims with aching agony, and sends them, often hobbling and decrepid to their doom.

"Gout, the child of luxury and excess, thrusts daggers through the joints, and racking with intensest pain, stiffens the members, distorts the limbs, or seizing some vital organ, wrings the life out.

"Consumption, thus vitiates the organic functions, blocks up with its chalky concretions, the vital passages, and sends its young, beautiful, and gifted victims, coughing, and parched with hectic fire, to a certain and early doom.

"Scrofula, poisons with its purulent virus, the living streams and with bulging tumors, deep scars and loathsome ulcers, disfigures, and melts down and wastes away the fair and goodly fabric.

"Cancer, that promethian vulture, a hungry parasite, feeding on the frame by piece-meal, tortures, and by slow, but certain inroads, finds the lurking place of life, and undermines it.

"Raving madness, that most terrific ill which flesh is heir to, replants its seeds of fire, to spring and burn afresh, in long generations.

"Moody melancholy, its sombre sister, stamps her heavy impress, to grow in sadness in succeeding minds.

"Idiocy, the child of incest, is renewed in horrid lineaments, in unmeasured succession.

"These are but a few of the physical agencies which, more or less, appreciably multiply the difficulties of comprehending man, as the object of medical administration.

"But we are not yet done with circumstances which most powerfully influence the organism in health and disease, and of vast value in practical medicine. We allude to mind, in its departments of intellect and passion.

"Too apt are we to forget, in the complaints of men, of physical ills, the powerful agency of mind, in their production and even in their cure.

"We forget while considering mind as a function of matter, that it is only technically so—that while (except it be in the recent spiritual revealings,) it is nowhere observed, but in connection with matter, yet it is superior to it, or at least, exerts what no other product of an organ does, a voluntary, reactive agency over, not only its own organ, but upon the whole sy tem. So powerful is this sometimes, as to enable man to sa authoritively, he will or he will not be sick and in accordance with this, resolves, succumbs to, or successfully repels disease.

"What is this agent? We know nothing of its essence. To mind, however, man owes his position among living creatures. This it is that originates, plans, and executes the schemes of life—this, that so distinguishes him by the display of powers, most wonderful—which renders him so prominently superior to all animals—this, that has led him from the wilds of barbarism, to the paths and refinements of civilized life,—this, which has elevated him to that throne of supremacy, before which all animated nature bows with reluctant, but coerced subserviency—this, in its moral department, which makes him a responsible agent, which claims for itself an independent life;—the something which "shrinks back on itself and shudders at destruction,"—this, which informs him that he shall never die,—this, which claims a right to rule and dictate the body in its outward actions and to govern its inward emotions,—this, when properly influenced by moral and religious principles, which raises him to heaven, allies him to angels, and unites him to his God; or when debased and subdued by animal nature, sinks him below the beasts that perish, and assimilates him to demons.

"Shall we then, in considering man as the subject of medical administration, fail to calculate the influence of this department of his constitution?—shall we forget his reasoning powers in disease, as medical philosophers, however, he may fail to exercise them, as he too often does, in swallowing the nostrums of empyrics, or confiding in pretenders? Certainly not. Much less, again, can we disregard his moral faculties, or considering him as a being possessed of passions and emotions."

We are also under obligations for a copy of the transactions of the New Hampshire Medical Society for 1855, from which we take a communication of Dr. G. W. Garland, of Lawrence, Mass., on the medical properties of the Yellow Jasmine, of Virginia.

### DR. GARLAND'S LETTER.

LAWRENCE, June 1, 1855.

*Dr. Peaslee:*

DEAR SIR:—When I saw you last I forgot to call your attention to the medical properties and uses of the Yellow Jasmine, or Jassamine, or Gelseminum Sempervерius, which grows wild in considerable abundance in Virginia. I think it will be classed among the nervines; still it is given in febrile diseases in from twelve to sixty drops, particularly in fever and ague, with three to five grains of quinine every two hours, till three doses are given. It affects some much more than others. Usually in twelve to twenty drop doses of the tincture a feeling is produced of *utter inability* to move, particularly the arms—still you *can* move. It is unlike any thing I ever felt. I took twenty-five drops sitting in my office, and when the operation came on I tried to think of some feeling to compare it with, and could think of nothing but the first dawn of consciousness after a full or complete syncope. It is a species of intoxication, which goes off leaving no headache or other unpleasant feeling.

In neuralgia, rheumatism and many uterine diseases, it has been found very efficacious. I have given the tincture in several cases of neuralgia with flattering results. The greatest benefit will be realized in the *periodical* type of that disease.

Have the goodness to call the attention of the Society at Concord to this medicine. Some one may know something more of its properties and uses. The bark of the root is the part used. Some prefer to use it with cornine instead of quinine—cornine, from Cornus Florida. When better understood it will be a remedy of great value and efficacy, I have no doubt.

Respectfully, yours,

G. W. GARLAND.

Since the above was prepared, the Introductory Address of John M. Watson, M. D., Professor of Obstetrics, &c., in the University of Nashville, has come to hand. Under the general head of the "Confounding of Many Things as a Characteristic of the Times," we find in a subdivision of "Science and Quackery most shamefully confounded," the following just and striking observations: "This topic, unlike those just disposed of, requires more than a passing notice. While the great benefactors of the age are laboring to investigate and disclose the great secrets of nature for the general good of mankind, there is a class who are working equally as hard to reduce a knowledge of their discoveries to quackish exclusiveness. These they take and box up, after having consolidated, colored, and shaped them into quack rotundies; or dilute them into strange fluids, with deceptive odors and lying labels. Were it practicable and profitable, the very earth itself would be rolled into pills, and the very ocean bottled and labelled by the numerous race of quacks. For so numerous have they become, that the man of science must feel the disagreeable and humiliating consideration, that while he is laudably and zealously engaged in scientific researches for the general and open good of others, he is at the same time subserving the interest of a race of quacks. He knows that his discoveries may pass into their hands, and be so transformed by their charlatanry, as to lose all their characteristics of identity; and in that way become available to them as new, potent and efficacious remedies.

"If a man were to purchase his neighbor's old horse for a mere trifle, and then fox, brand, and bishop him, so as to change his appearance and indications of age, and then sell him to another neighbor for a young and valuable horse, for ten times the amount which he gave for him, he would be regarded not only a jockey, but a swindler. The application is easy. A man buys some old, open, honest-faced article of medicine, and to use a jockey's phrase, foxes, brands, and bishops it, so as to destroy all external signs of its past appearances, and then sells it for one hundred times its cost; in so doing he certainly not only plays the part of a quack, but also of a knave. If we call one simple quackery, we must the other jockeyism. But neither of these terms reach the cases —one is swindling, and the other robbing—with but little distinction as to the acts themselves, or the terms by which they are expressed.

"Barnum's mermaid furnishes another good illustration of quackery: By means of the head, chest, and upper extremities of a monkey, and the inferior portion of a fish, he manufactured the semblance of an imaginary creature, which the fancies of many had long entertained. His factitious mermaid met their expectations, and reached their purses. His dupery for the time succeeded. So with the quack, by means of Calomel one part and Iodine another, he prepares a general panacea, the notion of which has been long cherished by many. This panacea, with all its pretensions, accords with their opinions, and is purchased by them at high rates, supposing as little that it is composed of Mercury and Iodine, as did Barnum's dupes that his mermaid was made of monkey and fish!

"I will add one more general illustration: When the sun rises and dispels the darkness of night, the nobler part of creation go forth to welcome him, and enjoy his blessings, especially the advantages of his light; but the owl-eyed, and wolf-eyed, skulk off to their dark holes and dens, that they may hide themselves and their prey from the light, and there await the forthcoming darkness of the night so congenial to their nature and pursuits. So, when the light of science is disseminated among us, the better part of men bid it welcome, and strive to enjoy its advantages openly, socially, and honestly, while others labor covertly, wickedly, and fraudulently to bring its revelations under the upas shade of quackry; for it has its poison as well as its darkness; its death's head, as well as its lying wonders; and its dark recesses as well as its sly showings.

"After all, there are some who think we ought to let quackery alone—let it take its way of darkness and deception undisturbed. Let us see if those of other callings are better than ourselves. Suppose that an irregular merchant, for instance, were to take a few common articles of merchandise, and change their names and aspects, then ascribe false qualities to them—all being duly certified and proven by a score of respectable names, improperly obtained—so as to enable him to sell them, at enormous rates, in the very midst of regular merchants, with an abundant supply of the same articles, bearing their proper names and appearances. Would they not expose, denounce, and hold up such a person to the just indignation of all honest men? Or, would the farmer be more charitable to his neighbor who might purchase his wheat of a common kind, at ordinary rates, and then sack it, and label it 'Wheat fresh from Egypt,' and then sell it, parcel by parcel, for one thousand times more than he gave?

"There can be no difference in principle, in the cases under consideration; the only difference is, that fraud is much more easily purchased in a traffic with medicines, than with merchandise or grain.

"Honorable medicine, in its open, regular way, can no more fraternize with quackery, than honesty with dishonesty, open dealing with knavery, or candor with deception. Our warfare against it is for life. We have been sworn at the altar of regular medicine, never to show it any professional quarters. Our heart, words, and influence will ever be put forth against it, professionally. How can we avoid it—meeting with it, as we do, constantly? For it has put forth its signs and traps almost everywhere. Her vile marks, almost as numerous as the frogs of Egypt, may be seen throughout the whole length and breadth of the land. We can scarcely open a newspaper without encountering some disgusting heading of a quack nostrum. Quack pamphlets have crept, unawares, into houses. Our almanacs lie worse about quack physic than they ever did about snow and frost. Our walls, gates, posts, and other fixtures, are defiled with its brands. Look out, your very hats and coat-tails are in danger of these vile marks."

---

### AMERICAN MEDICAL ASSOCIATION.

We desire to call the special attention of the profession among whom our Journal circulates, to the following communication, received from A. J. Semmes, M. D., Chairman of Committee, Coroner's Inquests, &c. This is an exceedingly important matter, and we hope that the profession will give the desired information and thus aid in bringing about some change in the present most absurd regulations upon this subject:

WASHINGTON, Dec. 10, 1855.

At the annual meeting of the National Medical Association of the United States, held at Philadelphia in May, 1855, the undersigned was appointed Chairman of a Committee to report to the Association "what measures should be adopted to remedy the evils existing in the present methods of holding Coroner's Inquests."

It being deemed desirable to obtain all facts, data, &c., relative to the objects of the commission, the undersigned would thankfully receive and acknowledge any documents or suggestions, such as the laws regulating inquests, post-mortems, examinations performed on a requisition of the legal authorities, the compensation of medical experts, the nature and tennure of the office of Coroner, etc.

A. J. SEMMES, M. D.,
Chairman of Committee on Coroner's Inquests.

---

The number of patients at the Charity Hospital for the month of November, 1855, says the New Orleans Medical News and Hospital Gazette, was as follows: admitted, 862; discharged, 798; died, 79. Births—males, 5; females, 7; stillborn, 1. Total, 13.

We learn that Leonard Marsh, M. D., has been elected to the chair of Latin and Greek in the University of Vermont, and has accepted the appointment.—*Boston Med. and Surg. Journal.*

*Newspaper Recommendations of Quack Medicines.*—We have so frequently taken occasion to state our unqualified disapprobation of any attempt to recommend, or bring into notice remedies whose composition is kept concealed from the profession, that it is with reluctance we again allude to so trite a subject. Our readers need not be told that no honorable physician will lend his influence to favor any such attempt. The Code of Ethics of the American Medical Association, and the laws of every respectable medical society throughout the world, condemn in the most unqualified terms all those concerned in such practices. The reason of this is obvious; if the composition of remedies is to be kept secret, there is an end of all improvement in therapeutics, a most important department of medical science. It may be said that the discoverer of a new remedy has as much right to the profits arising from its sale as a mechanic has to a patent for his invention, or an author to the copy-right of his book; and so far as the *legal* right goes, we presume that he has. But as a member of a liberal profession, which has for its object to relieve the sufferings of mankind, a physician has no moral right to do anything which can hinder the advancement of his profession in the art of curing or mitigating disease. Every improvement in medical science belongs to the profession, and not to the individual, "for if it is of real efficacy, the concealment of it is inconsistent with beneficence and professional liberality, and if mystery alone give it value and importance, such craft implies either disgraceful ignorance or fraudulent avarice."

It is with extreme regret that we notice in a newspaper of such high character and extensive influence as the Boston Daily Advertiser, a highly laudatory editorial notice of a secret nostrum, concerning which we have already made some remarks in a recent number of the Journal. We had hoped that the editors of a paper which lately took such high ground in respect to the independence of the press, would have the good sense to confine their criticisms to subjects within their own scope, and not endeavor to aid the sale of an alleged specific for half a dozen distinct diseases, concerning whose virtues they must be incapable of judging.

We do not make these remarks with any expectation of persuading the public of the absurdity of patronizing the "Peruvian Syrup," or any other empirical remedy. We are aware that our feeble voice will in no degree diminish the amount of its sale; would it were otherwise! It will doubtless become a fashionable medicine for a time, and having had its day, will become consigned to oblivion, to make way for some other nostrum. We cannot but complain, however, when we see an influential journal aiding a practice which is condemned by theunited voice of the profession. Nor shall we be thought to indulge the apprehension that the sale of such remedies is an injury to the practice of regular physicians; any one who is at all conversant with the matter knows that all medicines of this class are indirectly beneficial to the profession. The enormous amount of medicine indiscriminately swallowed by the public is a well-known cause of ill-health. It is as much for the pecuniary interest of the physician that the community should take quack-medicine, as it is for the lawyer that popular directions for making a will should be extensively circulated.—*Boston Med. & Surg. Jour.*

*The State Lunatic Asylum, Utica, N. Y.*—A writer in the Transcript says: This hospital occupies a commanding position just out of the city, and is a massive and imposing structure of stone. Opened to the public, January 16, 1843. It has been in operation nearly thirteen years, and has received not far from 5,000 patients. Formerly under the charge of Dr. N. D. Benedict, it is now superintended by Dr. John P. Gray, a gentleman eminently qualified for the position he holds, and well calculated both to win affection and command obedience from the unfortunate beings under his care.

About 1,000 patients were last year subjected to medical treatment, of whom more than 200 were discharged recovered and improved. The daily average attended to was 444.

The expenses amounted to $89,421 20, of which over $30,000 were appropriated to the culinary department alone. It is Dr. Gray's theory that his patients need a strengthening, nutritious diet, and all the amusements which convenience and propriety will admit. His intercourse with them unites in a marked manner gentleness and decision, sympathy and authority. It would be well for those interested in the treatment of insanity, to visit the Utica Asylum. They would be well paid for their trouble. If we may judge the benevolence of a State from the character and magnitude of its humane institutions, New York certainly deserves high eulogiums.—*Boston Med. Surg. Journal.*

---

### ERRATA.

It has been a source of no little annoyance and mortification to us, that the December number of our Journal should have gone out with so large a number of typographical errors, while we confess to some want of attention upon the part of those who usually correct the proof, it was in a good degree owing to confusion in the publishing office, connected with a change in the compositor's department, and to a great extent beyond our control. We fear the same difficulty will affect the present number, but think we can assure our subscribers that this is not to be a permanent characteristic of the publication, intending as we do, that its mechanical execution shall not be inferior to *any* of our exchanges.

